# The impact of social media use on tolerance, community peace, online ethical awareness among adolescents in the United Arab Emirates

**DOI:** 10.3389/fpsyg.2025.1500727

**Published:** 2025-02-14

**Authors:** Najia Al Ketbi, Mohammed Habes, Lubna Shaheen, Razaz Waheeb Attar, Dina Tahat, Amal Hassan Alhazmi

**Affiliations:** ^1^Mohamad Bin Zayed University for Humanities, Abu Dhabi, United Arab Emirates; ^2^Radio and TV Department, Faculty of Mass Communication, Yarmouk University, Irbid, Jordan; ^3^Unit of Policy, Planning, and Implementation, Department of School Education, Civil Secretariat, Quetta, Pakistan; ^4^University of Malaya, Kuala Lumpur, Malaysia; ^5^Management Department, College of Business Administration, Princess Nourah bint Abdulrahman University, Riyadh, Saudi Arabia; ^6^College of Education, Al Ain University, Al Ain, United Arab Emirates

**Keywords:** social media use on tolerance, social learning theory, community peace, online ethical awareness, media mindfulness

## Abstract

Social media use has increased after the COVID-19, indicating several effects on users. Talking specifically about its effects on adolescents, several studies have witnessed its positive and negative consequences on young users. This research also aims to examine the positive effects of social media use on adolescents from a Media Mindfulness Perspective, including tolerance, community peace, and online ethical awareness. Theoretically supported by Social Learning Theory, the data were gathered using 379 structured questionnaires. Results indicated an overall positive influence of social media use on adolescents in UAE. It is found that social media use positively affects tolerance among adolescents, suggesting an improved tolerance among the relevant users. Also, the effect of social media uses on community peace remained positive, implying that digital platforms are constructive means of teaching peace and harmony. Finally, results also indicated the positive effects of social media use on online ethical awareness among adolescents in UAE. Overall, these results suggest a positive, constructive role of social media use in changing the behaviors of the young generation. Under the social learning theory, this study also suggested social media as a powerful tool for learning positive behaviors, further influencing and improving societal peace and harmony. Finally, the study’s significance and limitations are discussed.

## 1 Introduction

Social media use has become a trending phenomenon across the globe. The primary motives behind its use are communication, information, education, and entertainment. The number of social media users is increasing daily, especially in a country like UAE, where digitalization is deemed an important aspect of everyday life (Hamad and Shehata, 2024). According to Kepios’s analysis, the number of internet users in UAE grew by 92,000 (a 0.9% increase) from January (Evli et al., 2023) to January 2024. By January 2024, UAE had 10.33 million internet users. At the beginning of 2024, the internet penetration rate in UAE was 91.0% of the total population (Ammari et al., 2024). This means that about 1.03 million people in UAE were not using the internet, accounting for 9.0% of the population who were still offline at that time. DataReportal’s data also shows that in January 2024, there were 6.38 million active social media user identities in UAE. This data also shows that roughly 2.58 million users are approximately 12 to 27 years old, suggesting 25% of Emirati adolescents are actively using social media (DataReportal, 2024). This increased social media use, especially among the Emirati adolescents, is consistent with the argumentation by Al Serhan and Elareshi (2019), considering it as impacting different aspects of life. As noted by Youssef and Al Malek (2023), social media plays a crucial role in people’s everyday lives and considerably impacts different aspects of their personal experiences. Platforms such as WhatsApp, Facebook, Snapchat, Instagram, and Twitter are critical global communication tools and can affect multiple areas of a person’s life. Social media also proposes the benefit of enabling users to share information, including content related to wellbeing and social harmony (Youssef et al., 2024). As a result, social media usually affects people’s attitudes, behaviors, and decision-making processes. Notably, today, the nature of online communication has altered how we interact, leading some to believe that obscuring behind a screen means their actions do not have the same ethical consequences. Despite this perception, social media is becoming increasingly influential in social and personal life (Elareshi et al., 2022; Scalvini, 2020). As ethical behaviors and values are learned through face-to-face interactions and experiences growing up, applying these principles online is important. Besides, social media helps young users learn about ethical behavior. For instance, transparency, a highly esteemed attribute in the professional world, should also be pursued in digital spaces (Kustiawan et al., 2023). Building genuine relationships and promoting community on social media depends on honesty. Being transparent with followers can improve trust and reputation, while deception can lead to losing reputation and violating perceived social ethics. As social media becomes more integrated into our personal and professional lives, learning and implementing a code of ethics is important for society, cultural betterment, and harmony (Bopp and Stellefson, 2020).

Thus, social media helps young users, especially young people, learn the importance of integrity, transparency, and respect in all forms of communication by facilitating ethical behaviors online (Habes et al., 2021; Kvalnes, 2019). However, current research also indicates a negative role of social media in aggravating intolerance and extremism among young generation that cannot be denied (Magis-Weinberg et al., 2021; Tarman and Kilinc, 2023; Wilf et al., 2023). For example Ul Ghafar et al. (2024) argued that the role of social media in promoting extremism and radicalization, as terrorist groups distort religious beliefs and promote ideologies that deviate from authentic religious teachings, cannot be denied. Also, social media encourages moral decline and facilitates the spread of sectarianism in many cases. Besides, it is also evident how young people are influenced by fictional characters from movies, TV shows, cartoons, and video games centered around conflict and violence. Platforms like Facebook and WhatsApp exacerbate this issue by rapidly spreading content that provokes aggressive reactions (Rauf, 2021).

Thus, considering the multifaceted role and impact of social media use on young users, this research aims to examine these effects from three perspectives: community peace and online ethical awareness among adolescents in UAE. Under the theoretical support of Social Learning Theory, this research aims to highlight the positive role of social media in Emirati society as an inevitable technological phenomenon. Notably, this research is important as social media use has drastically increased after COVID-19, indicating a significant technological gap in analyzing the current social media use and its impacts on Emirati society. The first section of current research highlights the topics, problems, aims, and gaps. The second part contains a brief review of existing literature. The third section justifies and discusses methodological approaches deemed suitable for current research. Finally, an analysis is conducted, followed by a brief discussion and limitations.

## 2 Literature review

### 2.1 Social learning theory

This research is supported by Social Learning Theory (SLT) by Albert Bandura, emphasizing the role and impact of social observation on the learning process of individuals. The relevant theory helps examine and conceptualize how certain behaviors are learned and imitated by people in a society. Based on the relevant study, social learning theory helps explain and relate the concepts of tolerance, community peace, and online ethical awareness to social media use. Especially among adolescents (Ammari et al., 2024), this theory helps explain these behaviors as important aspects of ensuring social mindfulness, peace, and harmony in Emirati society. It is notable that Emirati society is widely digitized, and social media use is increasing rapidly (Talib et al., 2021). In this context, social media has an important role in providing information, knowledge, communication, and learning from these platforms for Emirati.

**Figure 1:**
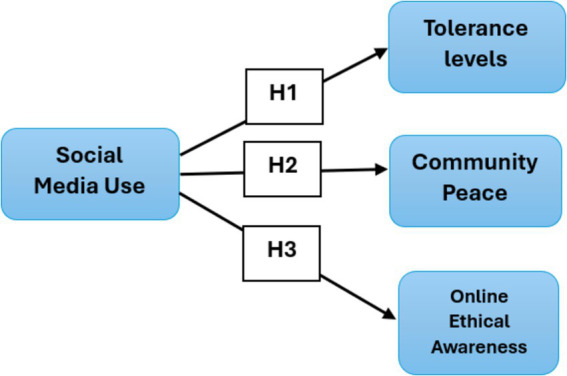
Conceptual framework of current research.

Consequently, these digital platforms’ role as a source of observation, learning, and imitation cannot be denied (Alodat et al., 2023) also attribute this social media to an increased social and political awareness among youngsters in Emirati. Accordingly, this theory is assumed by highlighting how individuals, particularly those in adolescents, can develop behaviors and attitudes through online interactions and experiences on social media websites (Sumadevi and Sumadevi., 2023). As adolescents engages with diverse content and communities online, they are exposed to different social norms, ethical standards, and communities online (Evli et al., 2023). These exposures can promote a greater understanding of tolerance, peace, and ethical behaviors by observing and internalizing positive examples shown by others in their networks (Jones et al., 2022). This theory highlights the prospect of social media as a robust tool for influencing and shaping the social and ethical development of young people as they learn from both the content they consume and their interactions with peers and influencers in the digital space (Fekih-Romdhane et al., 2024). Figure 1 illustrates the conceptualization of the current research study.

### 2.2 Social media and tolerance

Tolerance is the acceptance and respect individuals show toward diverse perspectives, beliefs, and cultures. The concept of tolerance helps measure how social media influences individuals’ openness and understanding toward different groups and their ability to engage respectfully with differing opinions (Baycar, 2023). Existing research indicates both positive and negative role of social media in spreading tolerance among young generation. For example, a study by Chandrima et al. (2020) conducted a study in Bangladesh, emphasizing the significant impact of social media in spreading extreme ideologies among youth. Many students need help to distinguish between authentic Islamic teachings and radical interpretations. A significant amount of online content focuses on the historical mistreatment of Muslims, portraying contemporary conflicts as extensions of Western dominance. This inability to differentiate between genuine religious principles and extremist views is rooted in cultural influences and exacerbated by social media. The Bangladeshi government and organizations are actively implementing initiatives to counter violent extremism, including leveraging social media to promote anti-violence campaigns. Besides, another study by Rauf (2021) examined how youth use social media and their attitudes toward violent extremism. The study indicated that young people generally preserve moderate perspectives on the issue. It determined key factors affecting these attitudes, including the quality of information, its perceived relevance, and ease of access. The results highlight that the way information is presented, along with its perceived value and usability, plays a key role in shaping youth attitudes toward violent extremism. Talking about the positive role of social media in improving tolerance, research shows that social networking sites (SNSs) can enhance people’s capability to socialize online and reduce feelings of loneliness. By providing more prospects for social networking, these platforms can increase self-esteem and create a sense of belonging, which improves the willingness to share personal experiences online (Cipolletta et al., 2020). This openness can positively affect overall wellbeing, as obtaining positive feedback from others on social media can amplify feelings of community integration and social support (Cipolletta et al., 2020). For example, adolescents use social networks to measure how others perceive them, to help confound shyness, and to build relationships. Those with less confidence in face-to-face interactions often prefer the online environment, where they may feel more secure (Wasiuzzaman and Edalat, 2021). This indicates that social media can foster social tolerance by allowing individuals to connect, share, and receive support in a way that might not be possible in offline settings (Dursun-Bilgin et al., 2018; Habes et al., 2022). Considering the cited literature, we assume that.

*H1*: Social media use positively affects tolerance levels among adolescents in the UAE.

### 2.3 Social media and community peace

Community peace is the level of harmony and cooperative coexistence within a community. This variable assesses how social media reduces conflicts, encourages understanding, and promotes a peaceful environment among community members (Hirblinger, 2023). Social media plays a crucial role in facilitating communication, raising awareness, and promoting dialog among communities, all of which are important components of peacebuilding, particularly among young users. It provides a platform for grassroots activism, allowing young people to organize nonviolent demonstrations, share practical information, and rally support for peace initiatives (Fournier-Sylvester, 2020). Social media also strengthens the voices of those who might otherwise go unheard, promoting inclusivity and diversity in peace efforts. Accordingly, social media positively impacts conflict resolution among young people by changing communication dynamics, strengthening diverse voices, and shaping public opinion. It accelerates information dissemination, promotes quick resolution of disputes, and encourages dialogue between conflicting parties (Yusoph and Yusoph, 2023). Besides, social media platforms provide a space for mediation efforts, allowing external mediators to engage with parties in conflict and encourage peaceful resolutions.

Notably, community peace efforts serve as a way for individuals to establish and sustain connections with others to promote the cause of peace, relying on “doing something together as part of groups’ doing something” (Brough et al., 2020). This collective sense of action contains the motivations behind the efforts and the resulting “emergent network,” which may seem to drive the actions themselves. These digital social networks shape individuals’ actions by “displaying for each other the meanings of their social situation” (Mujahid et al., 2021). For instance, in addressing issues such as gender and racial injustices, hashtags like #MeToo and #BlackLivesMatter have become potent symbols, integrating diverse participants into shared causes. These media practices enable many participants to form real or virtual groups, promoting solidarity and paving the way for civic engagement (Einstein et al., 2024). Through tools, i.e., hashtags, microblogs, or podcasts, individuals and organizations can collaborate to create innovative approaches to community peace and conflict resolution. In this context, peacebuilders analyze how peer-to-peer sharing of peace-related microblogs influences individuals’ experiences of peace. These processes emphasize the complex relationships between media users, their practices, and the resulting social realities (Gkora and Christou, 2023). Nonetheless, the possibility of spreading false information and increasing polarization can hinder constructive discourse and escalate conflicts (Zaky and Dilawati, 2024). Therefore, to effectively use social media for conflict resolution among the younger generation, it is important to execute strategies that decrease these risks and improve its role in facilitating empathy, understanding, and reconciliation (Wasiuzzaman and Edalat, 2021). Therefore, this study proposes that.

*H2*: Social media use positively affects community peace among adolescents in UAE.

### 2.4 Social media and ethical awareness

Online ethical awareness is an adherence to ethical standards in online interactions. It shows that social media influences individuals’ ethical decision-making and their ability to manage ethical dilemmas in virtual spaces, confirming responsible behavior online. In this regard, social media exposure to environmental and social issues has significantly increased over the past few decades (Gkora and Christou, 2023). Research shows that an increasing number of audiences are drawn to the principles of ethical consumerism (Brough et al., 2020). As these concerns acquire prominence, socially and environmentally conscious audiences are paying closer attention to the impact of mainstream societal practices. Generation Z, in particular, is acknowledged for its strong support of ethical initiatives. They actively participate in online advocacy, i.e., sharing government petitions and promoting direct action within their communities. The introduction of high-quality, ethical products and greater visibility for campaigns addressing human rights, political movements, environmental activism, and animal welfare has further amplified awareness and engagement (Rauf, 2021). According to Fournier-Sylvester (2020), social media is a powerful platform for the young generation to develop and acquire ethical awareness by exposing them to diverse perspectives, values, and behaviors. Young people can learn about different cultural norms and ethical standards through engaging with different communities and content, further broadening their understanding of right and wrong. As noted by Ali and Pasha (2024) today’s young generation is more aware of online bullying, privacy rights, and misinformation, leading them to stay more conscious about their actions. The interactive nature of social media allows for immediate feedback from peers and influencers, reinforcing positive behaviors and discouraging the harmful ones. This dynamic environment enables young users to reflect on their values and consider the impact of behavior on others, motivating a more profound sense of empathy and responsibility. For example, the importance of social networks is spacious, as they help reconnect family and friends, link organ donors with recipients, support different charity events, and increase the visibility of public figures (Rodriguez et al., 2021). Besides, social media platforms help young users to recognize ethical dilemmas, support social justice, and mental health awareness recognition. These all further highlight the importance of social media for the young generation today (Marin, 2022). Hence, this study proposes that.

*H3*: Social media use positively affects online ethical awareness among adolescents in UAE.

## 3 Research methods

This research employs a cross-sectional design (Gaus, 2017) and a closed-ended questionnaire to gather data. Cross-sectional studies are commonly preferred because they allow for quick data collection, improving the findings’ generalizability (Okada and Suto, 2003). The data is then scrutinized and coded to analyze using the SPSS (SPSS) and PLS-SEM.

### 3.1 Population and sampling approaches

The study population comprises Generation Z individuals aged 15–27. As noted earlier, around 2.58 million adolescents’ social media users are in UAE (DataReportal, 2024). The sample size for this study was calculated using Krejci and Morgan’s sample size calculator, which recommended a sample size of 384 respondents suitable for the current study (KENPRO, 2012). Surveys are distributed online through TGM Research UAE, which provides digital polling services to collect the data. Random sampling was used in this study to ensure that every participant had an equal chance of being selected, facilitating potential biases and increasing the generalizability of the results. The aim was to create a representative population sample, allowing for a more accurate and unbiased analysis of the relationships between social media use and the study variables (Acharya et al., 2013). The survey was available from June 2024 to August 28, 2024. By the end of this period, 379 responses were collected, resulting in a response rate of 98.6%. This rate is significantly higher than the adequate threshold of 60% (Holtom et al., 2022), supporting the reliability and generalizability of the study’s results.

### 3.2 Data gathering tool

The survey tool used in this study is a quantitative questionnaire formatted with a five-point Likert scale. The questions were adapted from previous research focused on tolerance, community peace, and online ethical awareness to ensure alignment with the study’s objectives. The first section of the questionnaire contains demographic information about the respondents, including age, gender, and locality. The second section consists of four statements related to social media use. The third section includes four items that measure the respondents’ tolerance, and the third section also contains four items measuring community peace levels. Finally, the fourth section has four statements to assess online ethical awareness among Generation Z in UAE. Table 1 provides a detailed overview of the survey sources and survey items.

**Table 1:** Questionnaire sources and survey items.

Variables	Sources	Survey items	# of items
Social media use	Habes et al. (2023a, 2023b)	Frequency of sharing posts on social media platforms (e.g., Facebook, Twitter, Instagram).	04
		Time spent interacting with social media daily (e.g., liking, commenting, messaging).	
		Engagement with controversial or polarizing topics on social media.	
		Use of social media for news consumption or seeking information on current events	
Tolerance	Ozimek et al. (2023)	Willingness to engage in discussions with individuals holding differing opinions on social media.	04
		Acceptance of diverse perspectives and minority views in online spaces.	
		Display respectful communication toward others, even during heated debates.	
		Avoidance of discriminatory or prejudiced content and interactions on social media.	
Community peace	Al Serhan and Elareshi (2019)	Use of social media to promote peaceful discussions around societal issues.	04
		Sharing messages or content that support conflict resolution and reconciliation in online communities.	
		Participation in community-building activities or peaceful campaigns on social media	
		Reduction of online conflicts through proactive intervention or moderation	
Online ethical awareness	Kalarani and Selvi (2021) and Scalvini (2020)	Recognition and reporting of unethical or biased content on social media platforms	04
		Critical assessment of the accuracy and reliability of information shared on social media	
		Commitment to uphold ethical standards in digital communication practices	
		Support for online policies and initiatives that promote ethical behavior and online safety	

## 4 Data analysis and findings

As data analysis in this research is based on Structural equation modeling, the process followed a two-step approach: First, the measurement model was evaluated to confirm its validity and reliability. The structural model was tested in the second phase, which included hypothesis testing and further analyses. First, the internal consistency between the study constructs is tested using convergent validity analysis (Chin and Yao, 2014). The relevant analysis shows that the factor loadings for all variables range between acceptable levels, with most item loadings above 0.5. The Average Variance Extracted (AVE) values for each variable indicate that a sufficient proportion of variance is captured by the constructs: Social Media Use (0.529), Tolerance (0.596), Community Peace (0.577), and Online Ethical Awareness (0.585). All variables’ Cronbach’s Alpha (CA) values are above the acceptable threshold of 0.7 (Brandt et al., 2016), indicating good internal consistency. Specifically, Social Media Use has a CA of 0.752, Tolerance has 0.736, Community Peace has 0.777, and Online Ethical Awareness is 0.779. Composite Reliability (CR) values for all variables also surpass 0.7 (Cowin et al., 2008), further establishing the reliability of the constructs, with Social Media Use at 0.798, Tolerance at 0.760, Community Peace at 0.813, and Online Ethical Awareness at 0.861. These findings confirm that the measurement model shows good convergent validity and reliability (see Table 2).

**Table 2:** Convergent validity analysis.

Variables	Items codes	Loads	AVE	CA	CR
Social media use	SM1	0.662	0.529	0.752	0.798
	SM2	0.545			
	SM3	0.639			
	SM4	0.756			
Tolerance	TOL1	0.524	0.596	0.736	0.760
	TOL2	0.705			
	TOL3	0.916			
	TOL4	0.950			
Community peace	CPE1	0.782	0.577	0.777	0.813
	CPE2	0.583			
	CPE3	0.768			
	CPE4	0.502			
Online ethical awareness	OEA1	0.499	0.585	0.779	0.861
	OEA2	0.517			
	OEA3	0.901			
	OEA4	0.561			

The goodness of fit is further assessed to examine the extent to which observed data fits well with the expected data (Van Vuuren, 2010). Thus, the goodness-of-fit results demonstrate that the model is well-aligned with the observed data. The SRMR values are exceptionally low, with 0.005 for the saturated model and 0.009 for the estimated model, indicating minimal discrepancies.

Similarly, the RMSEA values of 0.028 for the saturated model and 0.035 for the estimated model reflect a close fit within acceptable limits. The TLI values, at 0.947 and 0.971 for the saturated and estimated models, respectively, along with the CFI values of 0.935 and 0.950, confirm a strong model fit. While the NFI values (0.885 for the saturated model and 0.854 for the estimated model) are slightly below the ideal threshold of 0.90, they still indicate moderate improvement over a baseline model. The low Chi-square values (2.858 for the saturated model and 2.690 for the estimated model) further support the model’s validity. Collectively, these results affirm that the model is well-suited for further analysis (Sun, 2005). The results indicate that the model fits the data well and is fit for further analysis (see Table 3).

**Table 3:** Goodness of fit.

Goodness-of-fit measure	Saturated model	Estimated model	Threshold
SRMR (Standardized root mean square residual)	0.005	0.009	≤ 0.08
TLI (Tucker-Lewis index)	0.947	0.971	≥ 0.90
Chi-square (*χ*^2^)	2.858	2.690	>3.0
NFI (Normed fit index)	0.885	0.854	≥ 0.80
CFI (Comparative fit index)	0.935	0.950	≥ 0.90
RMSEA (Root mean square error of approximation)	0.028	0.035	≤ 0.06

The discriminant validity is further tested to determine the extent to which the study constructs are uncorrelated and have distinct values (Voorhees, 2016). As shown in Table 4, the Fornell-Larcker results show that each variable in the model is distinct from the others, confirming discriminant validity. For instance, the square root of the Average Variance Extracted (AVE) for Community Peace is 0.420, which is higher than its correlations with Online Ethical Awareness (0.061), Social Media Use (0.211), and Tolerance (0.317). Also, Online Ethical Awareness has a value of 0.534, greater than its correlations with other variables, such as Social Media Use (0.581) and Tolerance (0.545). Social Media Use has a value of 0.655, higher than its correlations with the other constructs. Tolerance shows a value of 0.544, which is higher than most of its correlations, although its correlation with Online Ethical Awareness (0.545) is close.

**Table 4:** Fornell-Larcker criterion.

	Community peace	Online ethical awareness	Social media Use	Tolerance
Community peace	0.420			
Online ethical awareness	0.061	0.534		
Social media use	0.211	0.581	0.655	
Tolerance	0.317	0.545	0.082	0.544

Further, the Heterotrait-Monotrait (HTMT) ratio results show that most of the relationships between the constructs meet the sufficient threshold for discriminant validity, typically below 0.90. The relationship between Online Ethical Awareness and Community Peace is very low, with an HTMT value of 0.006, indicating almost no relationship between these two constructs. Social Media Use and Community Peace have a moderate relationship with an HTMT value of 0.575, and Social Media Use and Online Ethical Awareness also show a moderate connection at 0.652. The relationships between Tolerance and Community Peace (0.239), Tolerance and Online Ethical Awareness (0.398), and Tolerance and Social Media Use (0.223) are relatively weak, suggesting adequate discriminant validity between these constructs (Cheung and Wang, 2017) (see Table 5). Altogether, the results show that the constructs in the model are distinct.

**Table 5:** Heterotrait Monotrait ratio scale.

	HTMT
Online ethical awareness ↔ Community peace	0.006
Social media use ↔ Community peace	0.575
Social media use ↔ Online ethical awareness	0.652
Tolerance ↔ Community peace	0.239
Tolerance ↔ Online ethical awareness	0.398
Tolerance ↔ Social media use	0.223

The coefficient of determination R^2^, also known as R-square analysis, is further tested to determine the predictive power of the independent variable (Social Media Use) (Renaud and Victoria-Feser, 2010). In this regard, the R-squared value of 0.506 suggests that approximately 50.6% of the variability in community peace can be explained by the predictors in the model for Community Peace. The adjusted R-squared value is identical at 0.505, indicating that the model fits the data well without substantial overfitting. For Online Ethical Awareness, the R-squared value of 0.337 indicates that the predictors account for 33.7% of the variability in online ethical awareness. The close-adjusted R-squared value of 0.336 indicates that the model apprehends a significant part of the variance. Finally, the Tolerance variable shows a very high R-squared value of 0.965, meaning that 96.5% of the variability in tolerance is well-explained by the predictors in the model. The adjusted R-squared value remains the same at 0.965, emphasizing an excellent fit and indicating that the model effectively highlights the underlying patterns for tolerance (see Table 6).

**Table 6:** Coefficient of determination R^2^.

	R-square	R-square adjusted
Community peace	0.506	0.505
Online ethical awareness	0.337	0.336
Tolerance	0.965	0.965

Finally, path analysis was used in this study to examine the relationships between social media use and key variables, including tolerance, community peace, and online ethical awareness. The results indicate significant positive associations between social media use and these factors. The coefficient for social media use on tolerance is especially 0.982, with a t-value of 24.276 and a *p*-value of 0.000, which shows a strong positive impact. Similarly, the relationship between social media use and community peace shows a coefficient of 0.711, a t-value of 9.462, and a p-value of 0.000, confirming a significant positive effect. Lastly, the effect of social media use on online ethical awareness is also validated with a coefficient of 0.595, a t-value of 7.711, and a p-value of 0.000, emphasizing its significant contribution. These findings suggest that social media promotes positive results in tolerance, community peace, and online ethical awareness (see Table 7).

**Table 7:** Hypotheses testing.

	Path coefficients	(M)	STDEV	*t-*value	*p*-values
Social media use → Tolerance	0.982	0.991	0.040	24.276	0.000
Social media use → Community peace	0.711	0.705	0.093	9.462	0.000
Social media use → Online ethical awareness	0.581	0.595	0.075	7.711	0.000

## 5 Discussion

This research examined the effects of social media on adolescents in the UAE. Notably, social media platforms’ rise, and overall use have significantly changed the social learning landscape in recent years. Researchers are now exploring how social media could offer more than just personal benefits; it could potentially enhance social behaviors among young users (Jones et al., 2022). Social media allows individuals to observe and engage with others’ actions, thoughts, and experiences in real-time across broad networks (Al Serhan and Elareshi, 2019). According to Al Serhan and Elareshi (2019), these platforms can enrich social experiences differently. They facilitate diverse forms of interaction, such as liking, sharing, and commenting on content, promoting a sense of virtual community and connection. Based on the proposition of social learning theory in the current study, Uwosomah (2021) argued that learning and changing existing perceptions and behaviors typically arise from interactions and experiences within one’s environment. Most interactions and communication now occur on social media, which can significantly affect adolescents’ perceptions and behaviors. Studies also indicate the use of social media has contributed to changes in the young generation’s social behavior. Increased reliance on social media has engaged the young generation with their local community, making virtual interactions with peers and family more enjoyable than in-person communication.

**Figure 2:**
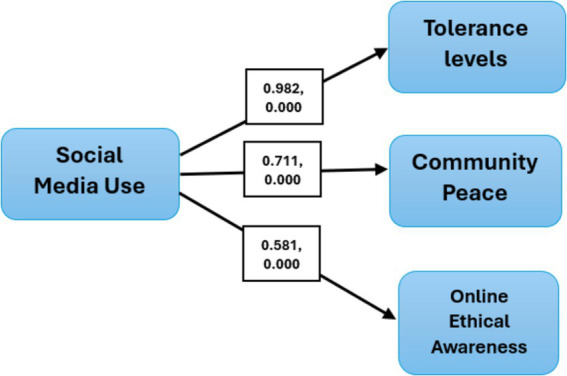
Results of path analysis (path and significance values).

Talking about the study results, all the hypotheses remained supported, indicating a positive, constructive role of social media among Emirati youth. Regarding the first hypothesis, “Social media use positively affects tolerance levels among adolescents in the UAE,” an overall positive effect of digital platforms is witnessed. Study respondents agreed with the importance of engaging in discussions with individuals with differing opinions on social media. They are willing to accept diverse perspectives and minority views, promoting an inclusive online environment. Besides, respondents emphasize the need for respectful communication, even during heated debates, underlining their commitment to maintaining positive interactions. Also, a shared understanding of avoiding discriminatory or prejudiced content and interactions promotes a more ethical approach to social media use. Bopp and Stellefson (2020) consider the conflicting opinion and acceptance on social media as a polarization of viewpoints. As noted opinion polarization can have positive outcomes, especially for young social media users. Users are exposed to a broader range of ideas and can appreciate different viewpoints by understanding diverse perspectives. Social media platforms facilitate this diversity by enabling users to interact with a vast network of people with varying opinions, fostering a more inclusive environment. Research has shown that social media usage contributes to developing networks with heterogeneous views, where discussions often include aligned and divergent perspectives (Alshare et al., 2019; Brough et al., 2020; Tetep et al., 2020). As such, social media plays a significant role in promoting understanding, empathy, and the positive impacts of diversity, helping young users manage and appreciate the complexities of contemporary communication (Figure 2).

Regarding the second hypothesis, “Social media use positively affects community peace among adolescents in UAE,” the study respondents agree with using social media to promote peaceful societal discussion. They believe in sharing messages that support conflict resolution and reconciliation within online communities. Besides, respondents are engaged in community-building activities and peaceful campaigns on social media. They also emphasize the significance of reducing online conflicts through proactive intervention and moderation, showing a collective effort toward promoting a more harmonious online environment. Consistent with these findings Evli et al. (2023) argued that social media encourages social interactions and affects global social behaviors, creating possibilities for acceptance behaviors and growth (Fekih-Romdhane et al., 2024). Social behavior, which encompasses attitudes, perceptions, and reactions toward others, is affected by different factors, including social media (Blandi et al., 2022; Vizcaya-Moreno and Pérez-Cañaveras, 2020). Current research has also shown that social media positively impacts youth by promoting online tolerance, community peace, and ethical behavior. In line with these results, studies by Villamil and King (2024) and Ozimek et al. (2023) have examined how social media affects group behavior and academic performance and also indicated the positive role of social media.

Finally, analysis also supported the third hypothesis “Social media use positively affects online ethical awareness among adolescents in UAE.” Respondents indicated a commitment to recognizing and reporting unethical or biased content on social media platforms. They emphasized the significance of critically evaluating the accuracy and reliability of information shared online, ensuring that only credible and ethical content is consumed. Also, there was a clear agreement on the necessity of upholding ethical standards in digital communication, highlighting the need for respectful and responsible interactions. Respondents also supported adopting online policies and initiatives to promote ethical behavior and improve online safety, promoting a safer and more accountable social media environment. Similarly, Adegboyega (2019) examined its influence on youth behavior. These studies also indicated the positive effects of social media use on young generation’s behaviors in different dimensions. A study by Alshare et al. (2019) also examined the impact of social media on the moral and social behavior of students at Jordanian universities. Data gathered from 1,000 students indicated that social media significantly impacts the development of moral and social attitudes among students. Besides, there were notable differences in responses based on gender, age, academic level, and English language proficiency. These variations suggest that social media affects students differently based on individual characteristics.

However, despite these positive effects witnessed by the cited literature and current study, the negative effects, such as binge usage (Aksar et al., 2023), cyberbullying (Pasha et al., 2022), exposure to indecent content (Ali and Pasha, 2024), and others, cannot be denied. Despite these negative effects and consequences (Adegboyega, 2019; Viţelar, 2019), this research provided a robust picture of its positive role and impact on Emirati society. Thus, this research provided useful insights into various stakeholders. For researchers, it provided a thorough understanding of how social media influences ethical behaviors and community values in a rapidly changing digital landscape (Duffett, 2017).

## 6 Conclusion

This research highlights the significance of maintaining integrity, fostering tolerance, and promoting responsible communication on social media platforms. As social media continues to play a prominent role in modern communication and information sharing, it becomes critical to address the ethical challenges that arise from its widespread use. This study provides insights into how these dimensions influence user behavior and online experience. Policymakers and regulatory bodies can use these results to develop well-informed guidelines and policies encouraging positive digital engagement. Such policies should improve user awareness of ethical standards and promote responsible use of social media. Promoting a more inclusive digital environment can also help mitigate issues such as selective exposure, misinformation, and online conflicts. Therefore, it is concluded that social media plays a powerful role in shaping how adolescents experience tolerance, community peace, and online ethical awareness. Young users can build a more inclusive digital environment through deferent communication and exposure to diverse perspectives. By promoting ethical behavior and encouraging positive interactions, social media empowers adolescents to engage thoughtfully and constructively in online spaces.

### 6.1 Limitations and recommendations

This research presents several limitations that should be addressed in future studies. First, the study was conducted in the UAE, which may limit the generalizability of the findings to other geographical regions with diverse cultural, social, and political contexts. The results may need to fully capture the complexities of how social media impacts adolescents in different settings. Second, using a single-method design, particularly Structural Equation Modeling (SEM), constrains the scope by not accounting for qualitative perspectives or mixed-method approaches. While useful for comprehending relationships between variables, SEM does not establish causality, and its reliance on quantitative data limits the depth of insights.

Third, the study focused solely on tolerance, community peace, and online ethical awareness as outcomes of social media use. This narrow scope restricts a comprehensive exploration of the broader impacts of social media, such as emotional wellbeing, political engagement, or mental health. Future researchers should consider incorporating diverse research methods, i.e., qualitative interviews or case studies, to address these limitations and provide richer, contextual insights. Besides, mixed method approaches that combine qualitative and quantitative data could enhance understanding by indicating deeper social dynamics. Expanding the scope to include a variety of outcomes beyond tolerance, community peace, and online ethical awareness will also provide a more holistic view of social media’s impact. Also, future studies should aim to conduct comparative analyses across different regions to evaluate the broader applicability of the findings. By addressing these limitations, future research can offer a more in-depth and comprehensive understanding of social media’s effects on adolescents.

## Data Availability

The original contributions presented in the study are included in the article/supplementary material, further inquiries can be directed to the corresponding author.
